# Supplementary material for: mSWI/SNF complex inhibition sensitizes KRAS-mutant lung cancers to targeted therapies via epithelial-mesenchymal subversion
Source: bioRxiv. 2026 Mar 1:2026.02.27.708377. Preprint. [Version 1] doi: 10.64898/2026.02.27.708377 (PMC13160128; doi:10.64898/2026.02.27.708377)
Supplement: Supplement 1 [file NIHPP2026.02.27.708377v1-supplement-1.pdf]

bioRxiv preprint doi: https://doi.org/10.64898/2026.02.27.708377; this version posted March 1, 2026. The copyright holder for this preprint (which was not certified by peer review) is the author/funder, who has granted bioRxiv a license to display the preprint in perpetuity. It is made

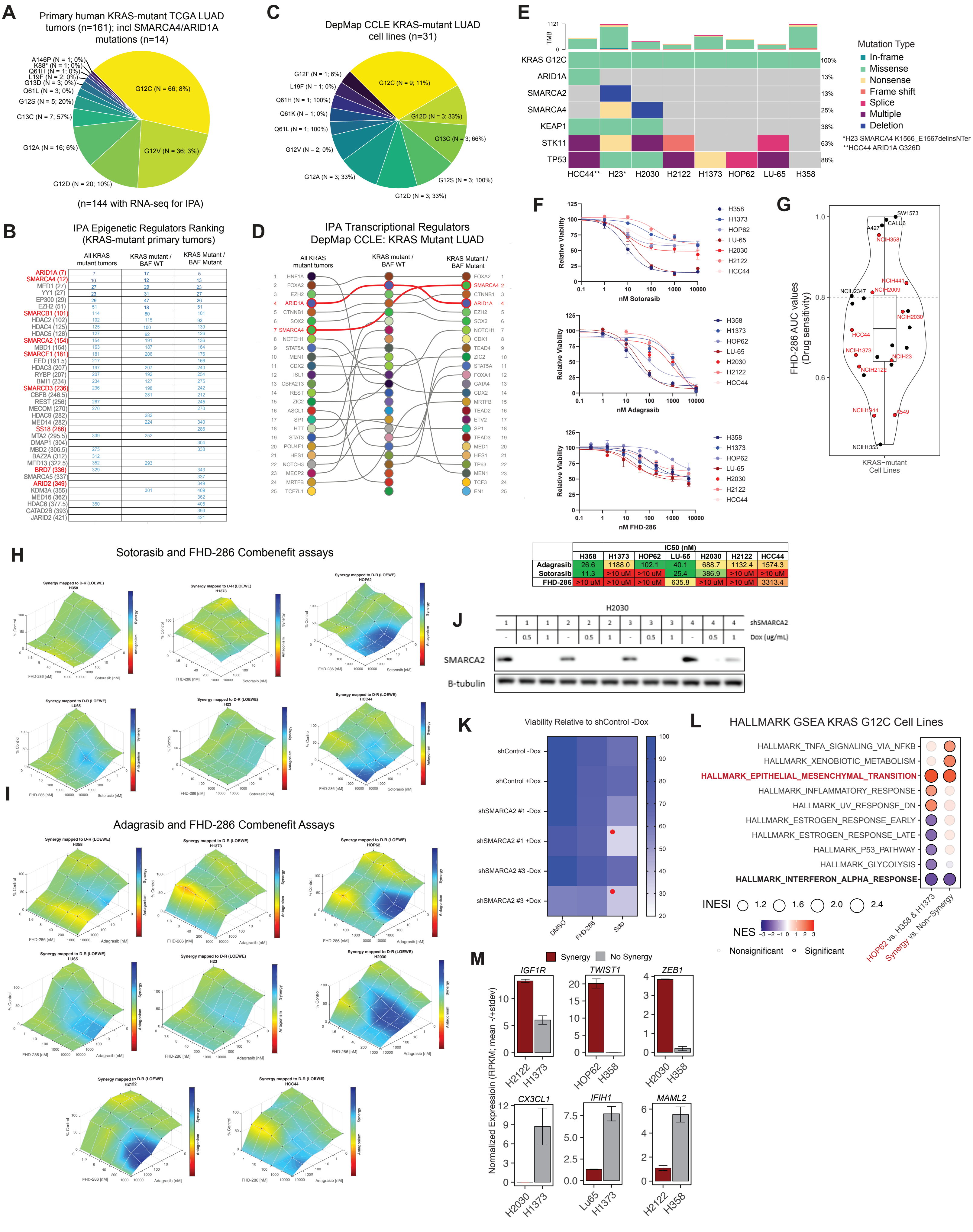

	IC50 (nM)						
	H358	H1373	HOP62	LU-65	H2030	H2122	HCC44
Adagrasib	26.6	1188.0	102.1	40.1	688.7	1132.4	1574.3
Sotorasib	11.3	>10 uM	>10 uM	25.4	386.9	>10 uM	>10 uM
FHD-286	>10 uM	>10 uM	>10 uM	635.8	>10 uM	>10 uM	3313.4

bioRxiv preprint doi: https://doi.org/10.64898/2026.02.27.708377; this version posted March 1, 2026. The copyright holder for this preprint (which was not certified by peer review) is the author/funder, who has granted bioRxiv a license to display the preprint in perpetuity. It is made available under aCC-BY 4.0 International license.

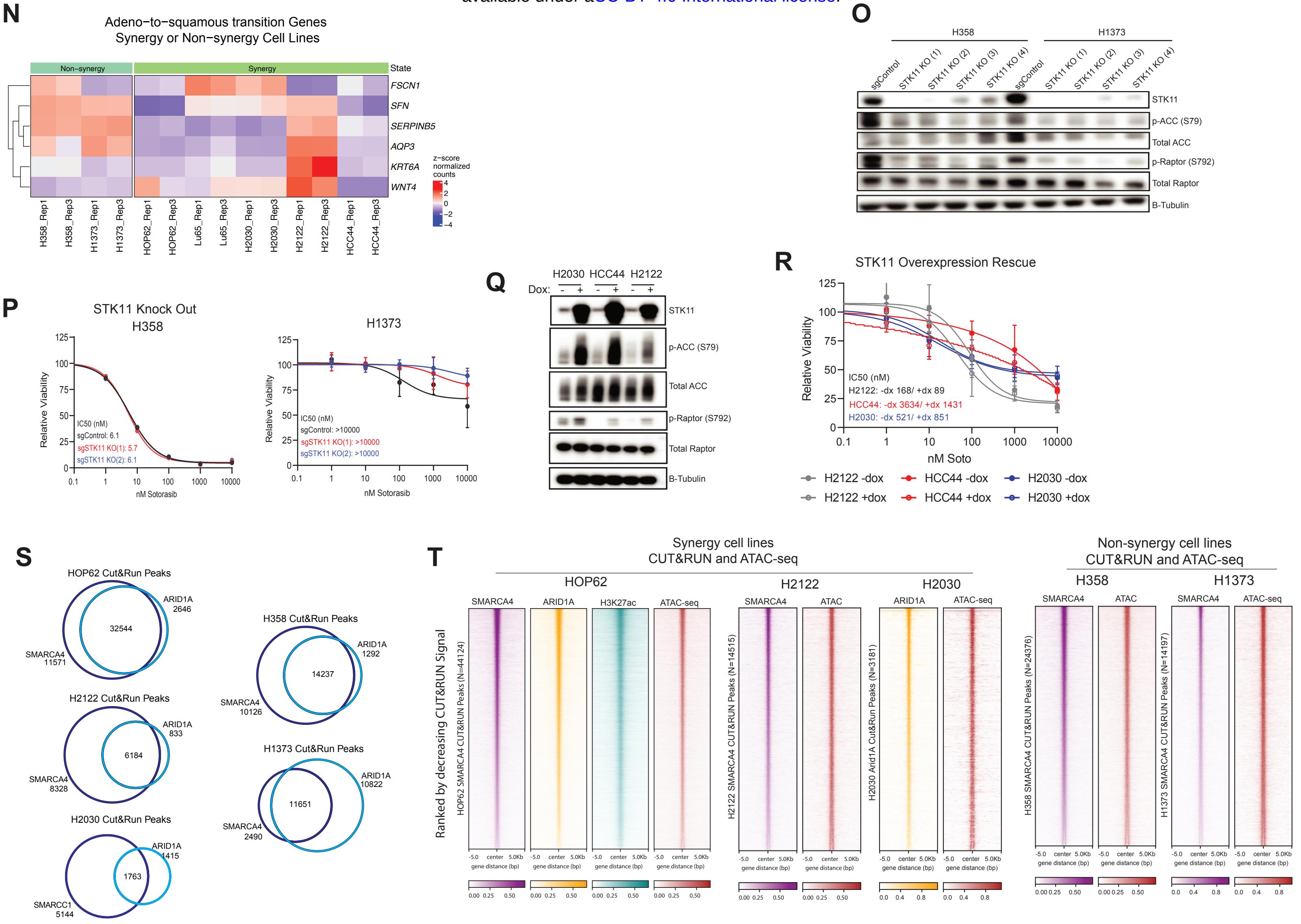

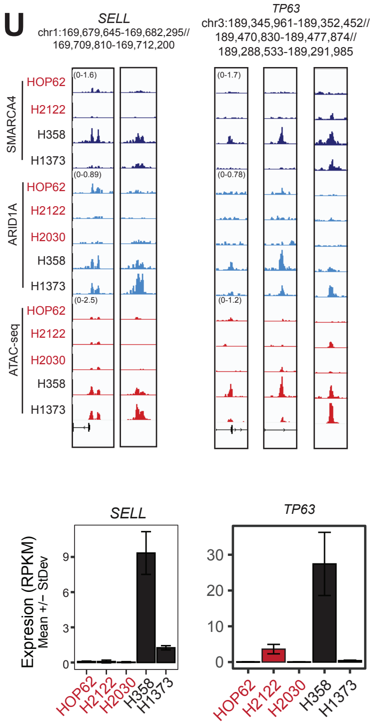

Figure S1. KRAS G12C-mutant cell lines are distinguished by specific mSWI/SNF chromatin features. A. Pie chart of KRAS mutations in TCGA LUAD primary tumors (total n=161, n=142 used in Fig1A IPA analysis). Samples include those with ARID1A and SMARCA4 mutations. B. Summary of ranks for epigenetic upstream transcriptional regulators (IPA) in KRAS-mutant LUAD primary tumors with and without BAF mutations; mSWI/SNF factors are highlighted. C. Pie chart of KRAS mutation type in CCLE cell lines (n=31). D. Results of upstream transcriptional regulator analysis in CCLE cell lines, analyzed by BAF mutation status. E. Mutation status of ARID1A, SMARCA4, STK11, KEAP1, and TP53 in KRAS-mutant cell lines used in this study. F. IC50 curves of sotorasib, adagrasib, and FHD-286 treatments across cell lines indicated. Table of IC50 values shown below. G. PRISM (Broad Institute) FHD-286 drug sensitivity AUC values for KRAS mutant cell lines; cell lines used in this study are shown in red; AUC>0.8 denotes FHD-286 sensitive lines (see also Table S1). H-I. Combenefit synergy studies performed at t=96 hours using (I) sotorasib and (J) adagrasib in combination with FHD-286. J. Immunoblot of SMARCA2 levels following dox induction of SMARCA2 shRNAs in H2030 cells. B-tubulin is loading control. K. Viabililty plot of SMARCA2 shRNA H2030 cells following DMSO, FHD-286 or sotorasib treatment. Viability measured relative to shControl with no dox treatment. Substantial decreases in cell viability upon SMARCA2 knock down and sotorasib treatment are indicated by red dots. L. Hallmark GSEA performed on HOP62 versus H358 and H1373 cell lines and all synergy versus non-synergy cell lines. M. Bar graphs indicating normalized expression values (RPKM) for top synergy- or non-synergy- specific genes. N. Gene signature for adenocarcinoma-to-squamous cell carcinoma in KRAS-mutant cell lines (REF) using biological replicates of RNA-seq. O. Immunoblot of STK11, phosphorylated and total ACC, phosphorylated and total RAPTOR levels upon knock out (KO) of STK11 in H358 and H1373 cells. B-tubulin is loading control. P. IC50 curves for sotorasib of H358 and H1373 cells upon STK11 KO. Q. Immunoblot of STK11, phosphorylated and total ACC, phosphorylated and total RAPTOR levels upon dox induction of STK11 over-expression in H2030, HCC44 and H2122 cells. B-tubulin is loading control. R. IC50 curves for sotorasib of respective cell lines upon over-expression of STK11. S. Venn diagrams showing overlap between mSWI/SNF CUT&RUN peaks for SMARCA4 or SMARCC1 and ARID1A across all cell lines. T. Heatmaps of SMARCA4 or ARID1A (CUT&RUN) occupancy and ATAC-seq in synergy and non-synergy cell lines. H3K27ac CUT&RUN correlates with BAF occupancy and ATAC signal in HOP62 cells. U. Top, Representative tracks at the SELL, and TP63 loci showing mSWI/SNF complex occupancy (ARID1A and SMARCA4 CUT&RUN) and ATAC-seq; bottom, RNA-seq normalized expression (RPKM) bar plots of SELL and TP63.

Gentile, Feng et al. 2026

bioRxiv preprint doi: https://doi.org/10.64898/2026.02.27.708377; this version posted March 1, 2026. The copyright holder for this preprint (which was not certified by peer review) is the author/funder, who has granted bioRxiv a license to display the preprint in perpetuity. It is made available under aCC-BY 4.0 International license.

Figure S2

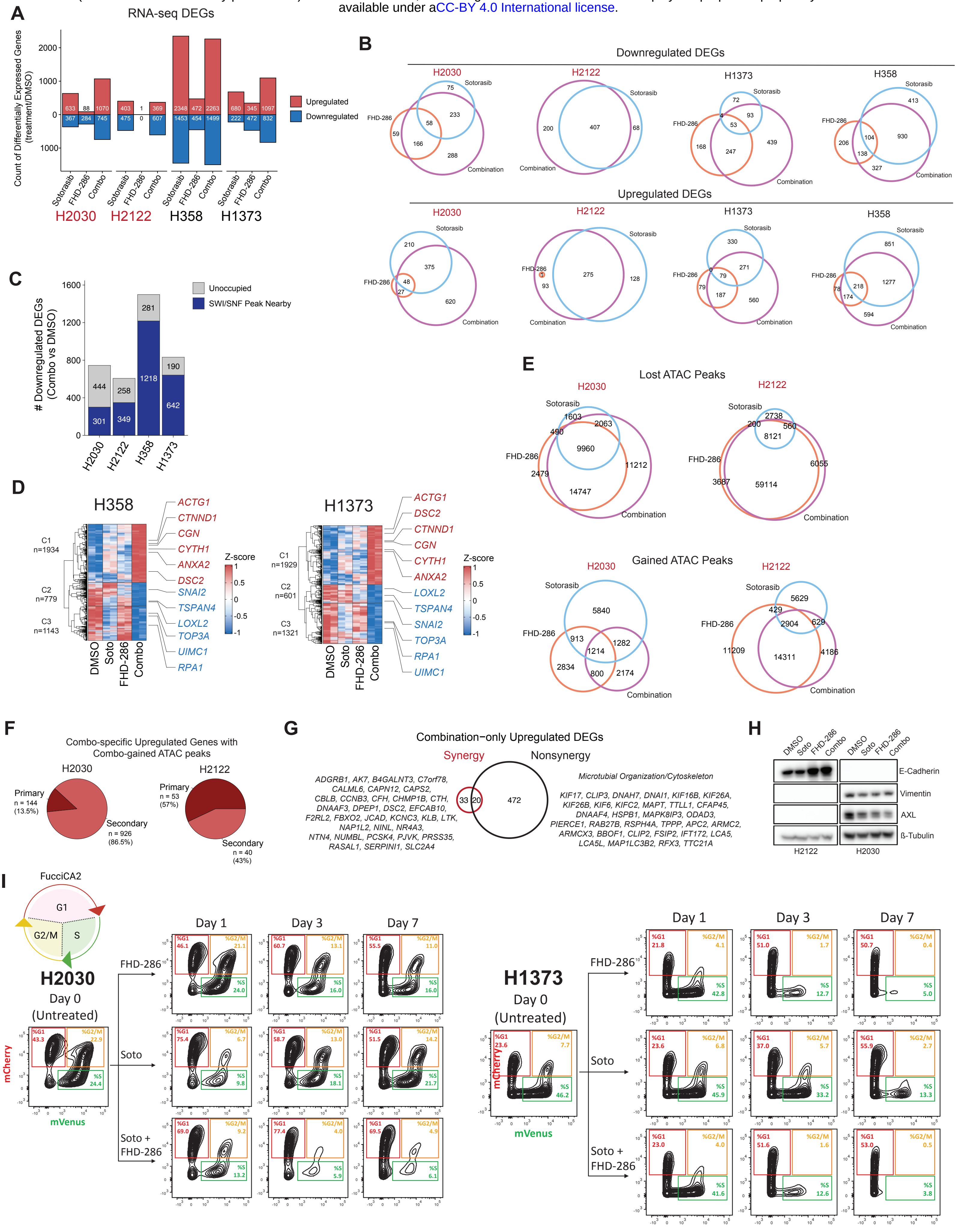

bioRxiv preprint doi: https://doi.org/10.64898/2026.02.27.708377; this version posted March 1, 2026. The copyright holder for this preprint (which was not certified by peer review) is the author/funder, who has granted bioRxiv a license to display the preprint in perpetuity. It is made available under aCC-BY 4.0 International license.

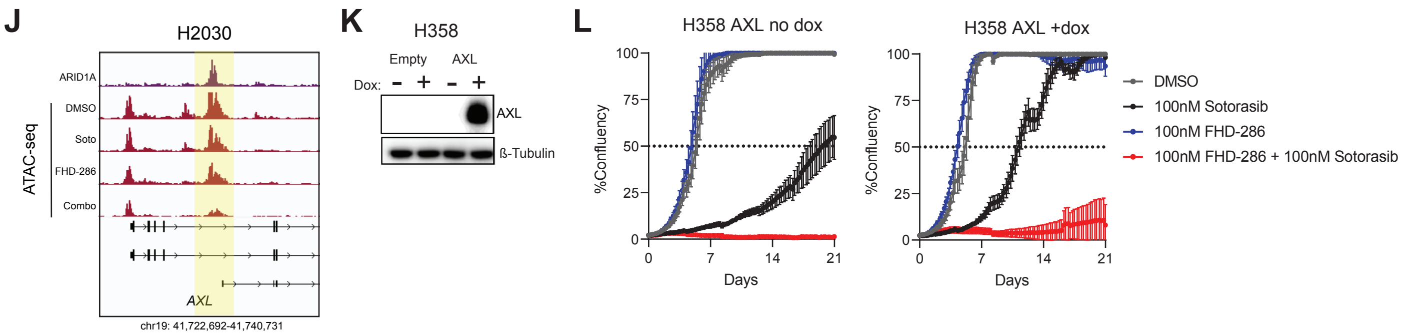

Figure S2. Targeted inhibition of mSWI/SNF complexes in combination with sotorasib treatment impacts EMT and cell cycle across KRAS-mutant cell lines. A. Barplots summarizing the number of differentially expressed genes in single and combination drug treatments in synergy and non-synergy cell lines. **B.** Euler diagrams summarizing the number of upregulated or downregulated genes shared between sotorasib, FHD-286 and combination sotorasib and FHD-286 treatments in each cell line. **C.** Barplots summarizing the proportions (with count summaries) of downregulated genes in the combination treatment with mSWI/SNF occupancy in each cell line. **D.** Heatmaps of top 10% deregulated genes upon combination treatment in H358 and H1373 cells. Gene expressoion values were row z-scored normalized. Genes of interest are highlighted in red (up-regulated) or blue (down-regulated). **E.** Euler diagrams summarizing the number of shared and specific lost or gained ATAC-seq chromatin accessible sites following sotorasib, FHD-286 and combination Sotorasib and FHD-286 treatments in each cell line. **F.** Pie charts representing percentage of combination specific upregulated genes with concordant chromatin accessibility (ATAC-seq) changes upon combination treatment (primary targets) in H2030 and H2122. Nearest ATAC-seq peak was assessed within a 30kb window of TSS. **G.** Venn diagram overlap of combination only up-regulated genes in synergy and non-syngery cell lines (resulting from subtracting sotorasib up-regulated genes). Relevant genes are listed. **H.** Immunoblot of EMT markers E-Cadherin, vimentin, and AXL upon sotorasib, FHD-286 or combination drug treatments in H2030 and H2122 cells. B-tubulin is the loading control. **I.** Florescent Ubiquitination-based Cell Cycle Indicator (FUCCI) reporter system in H2030 and H1373 cells following respective treatments at Day 1, 3 and 7. **J.** Representative tracks at the AXL locus in H2030 cells, showing mSWI/SNF complex occupancy (ARID1A CUT&RUN) and chromatin accessibility (ATAC-seq) across treatment conditions. Loss of ATAC signal is highlighted in a yellow box. **K.** Immunoblot of AXL protein levels in H358 cells following 21 days of dox inducible AXL expression. B-tubulin is loading control. **L.** Cell proliferation curves of H358 cells without AXL overexpression (no dox) and with AXL overexpression (+dox) treated with DMSO, sotorasib, FHD-286 or combination following 21 days of treatments.

bioRxiv preprint doi: https://doi.org/10.64898/2026.02.27.708377; this version posted March 1, 2026. The copyright holder for this preprint (which was not certified by peer review) is the author/funder, who has granted bioRxiv a license to display the preprint in perpetuity. It is made available under aCC-BY 4.0 International license.

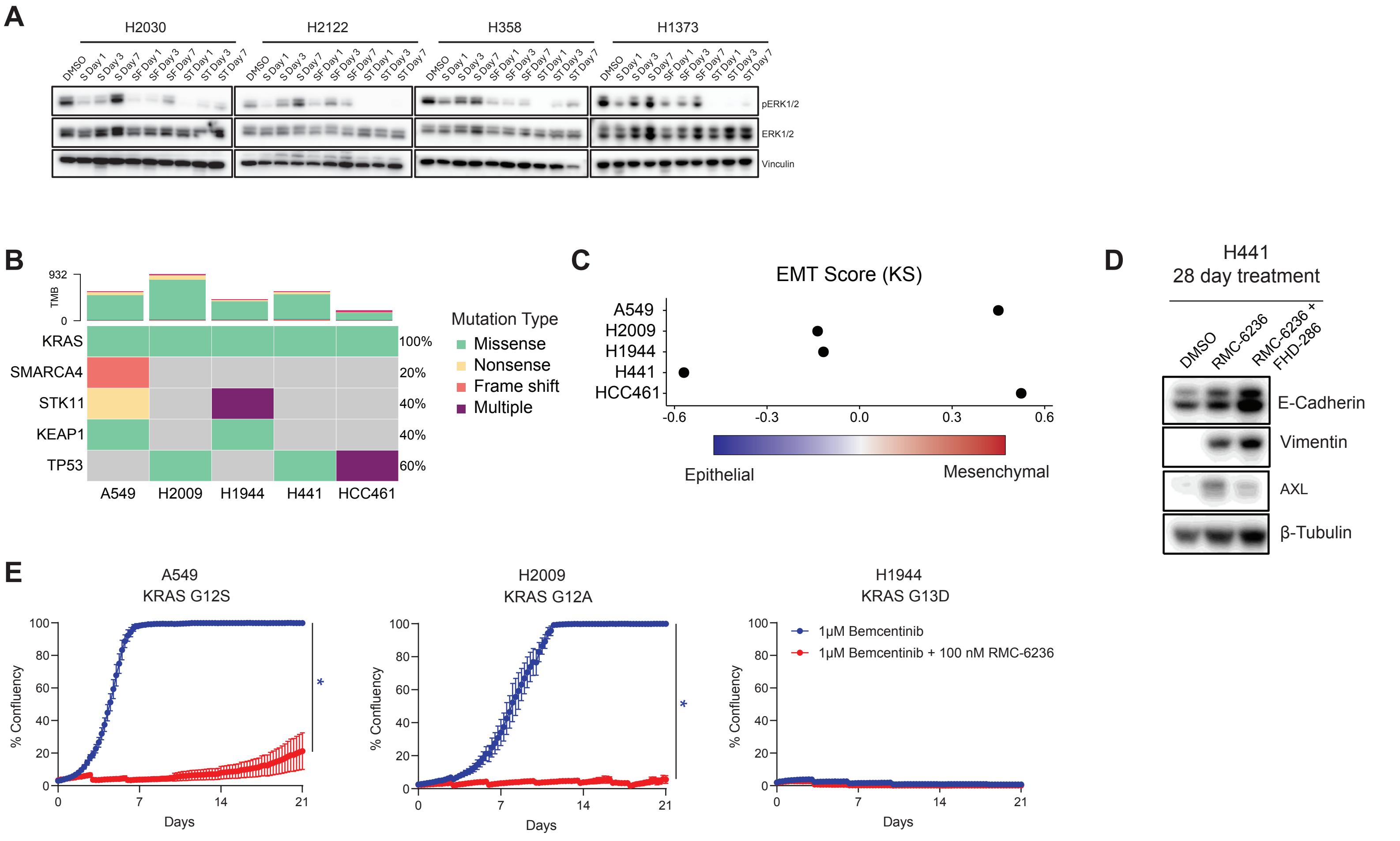

Figure S3. FHD-286 sensitizes non-G12C KRAS-mutant lung cancer models to targeted therapies.
A.Western blot of cells treated with DMSO, 100 nM sotorasib (S), 100 nM sotorasib + 100 nM FHD-286 (SF), or 100 nM sotorasib + 30 nM trametinib (MEK inhibitor) (ST) at 1, 3 and 7 days. B. Mutation status of SMARCA4, STK11, KEAP1, and TP53 in non-G12C KRAS-mutant cell lines. C. Epithelial to mesenchymal transition (EMT) score for non-G12C KRAS-mutant cell lines using the KS (Kolmogorov-Smirnov) method. D. Immunoblot of H441 cells treated with 100 nM RMC-6236 for 28 days leading to induction of AXL, which can be blunted by co-treatment with 100 nM FHD-286. E. Confluence of cells exposed to the indicated treatments was measured once every 6 hours over 21 days. Media was replenished every 4 days. Data represents mean +/-SEM of n=4 replicates. Welch's t-test was used to compare groups at endpoint. *$p < 0.05$.

bioRxiv preprint doi: https://doi.org/10.64898/2026.02.27.708377; this version posted March 1, 2026. The copyright holder for this preprint (which was not certified by peer review) is the author/funder, who has granted bioRxiv a license to display the preprint in perpetuity. It is made available under aCC-BY 4.0 International license.

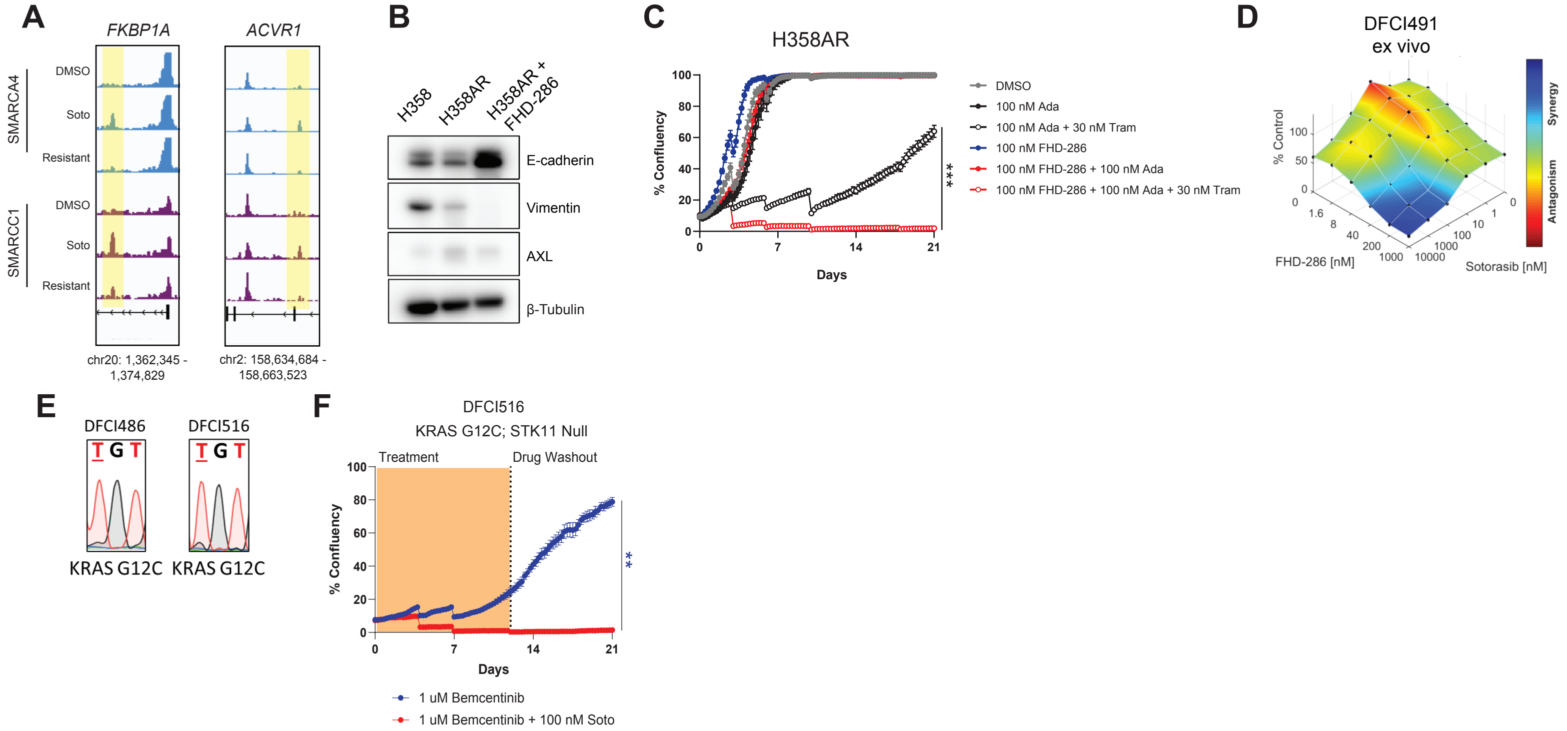

Figure S4. mSWI/SNF inhibition re-sensitizes patient-derived organoid models to KRAS inhibitors.
A.IGV track examples of SMARCA4 and SMARCC1 CUT&RUN peaks across DMSO, soto (24hr) and resistant conditions in H358 cells at the *FKBP1A* and *ACVR1* loci. Increased peaks of interest are highlighted. B. Western blot comparing expression of EMT markers in H358, H358AR (in presence of 100 nm adagrasib), and H358AR (in presence of 100 nM adagrasib) treated with 100 nM FHD-286 for 96 hrs. C. Following 1 week adagrasib washout, H358AR cells were challenged with the indicated treatments. Confluence was measured once every 6 hours over 21 days, with media replenished every 4 days. D. DFCI491 ex vivo tumor spheroids were challenged with drug combination matricies with dose titrations of sotorasib and FHD-286 in ULA 384-well plates. Following 96 hours, 3D CTG assay was performed and drug synergy was calculated using Combenefit software. Processed tumor material 40-100 μm was frozen and used for experimental repetitions. One representative experiment out of three is shown. E. Sanger sequencing results for newly established DFCI486 and DFCI516 patient-derived cell lines confirms retention of KRAS G12C mutation. F. Confluence of DFCI516 cells challenged with AXL-specific inhibitor (bemcentinib) alone or in combination with sotorasib. One-way ANOVA was used to compare groups at endpoint. **p < 0.005. Data represent mean +/- SEM of n=4 replicates.

bioRxiv preprint doi: https://doi.org/10.64898/2026.02.27.708377; this version posted March 1, 2026. The copyright holder for this preprint (which was not certified by peer review) is the author/funder, who has granted bioRxiv a license to display the preprint in perpetuity. It is made available under aCC-BY 4.0 International license.

A

Organoid Model [1]	XDO_PHLC239	XDO_PHLC194	XDO_PHLC344	XDO_PHLC207
KRAS mutation	p.G12C	p.G12C	p.G12C	p.G12C
Co-mutations & copy number alterations [2]	ALK amplification; NF2 p.Q362* shallow deletion;	KEAP1 p.P278A; MYC amplification; SMARCA4 p.M1305Ifs*53	TP53 p.G199V; RB1 p.X654_splice.	STK11 p.G155=, p.X155_splice; RB1 splice; BRCA2 p.E115*

PDX Model [1]	PDX_PHLC239	PDX_PHLC194
KRAS mutation	p.G12C	p.G12C
Co-mutations & copy number alterations [2]	NF2 p.Q362* deep deletion; CDKN2A deep deletion	KEAP1 p.P278A; BRCA2 p.E953K*; MYC amplification; SMARCA4 p.M1305Ifs*53
In vivo response to KRAS G12Ci [3]	resistant	sensitive

1 Mirhadi S, Tam S, Li Q, Moghal N, Pham NA, Tong J, Golbourn BJ, Krieger JR, Taylor P, Li M, Weiss J, Martins-Filho SN, Raghavan V, Mamatjan Y, Khan AA, Cabanero M, Sakashita S, Huo K, Agnihotri S, Ishizawa K, Waddell TK, Zadeh G, Yasufuku K, Liu G, Shepherd FA, Moran MF, Tsao MS. Integrative analysis of non-small cell lung cancer patient-derived xenografts identifies distinct proteotypes associated with patient outcomes. Nat Commun. 2022 Apr 5;13(1):1811. doi: 10.1038/s41467-022-29444-9. PMID: 35383171; PMCID: PMC8983714.

2 Queried gene list = { STK11, KEAP1, TP53, MYC, RB1, PTEN, BRCA1, BRCA2, NF1, NF2, CDKN2A, SMARCA4, SMARCA2, SMARCA1, ARID1A, EGFR, ALK, ROS1, RET, MET, ERBB2, NRG1, BRAF}; variants of unknown significance are not shown. Note: truncating mutations in SMARCA2/4 with predicted loss of function.

3 Rosen JC, Cao P, Pham NA, Waas M, Li Q, Hueniken K, Wang M, Navab R, Wybenga-Groot L, Radulovich N, Niedbala M, Koers A, Ross S, Moran MF, Sacher A, Tsao MS. Modeling response to the KRAS-G12C inhibitor AZD4625 in KRASG12C NSCLC patient-derived xenografts reveals insights into primary resistance mechanisms. Br J Cancer. 2026 Jan;134(1):165-174. doi: 10.1038/s41416-025-03216-w. Epub 2025 Oct 11. PMID: 41076489; PMCID: PMC12765001.

B

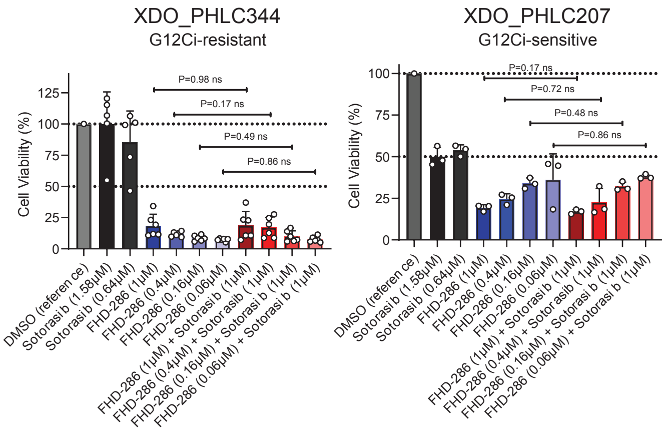

C

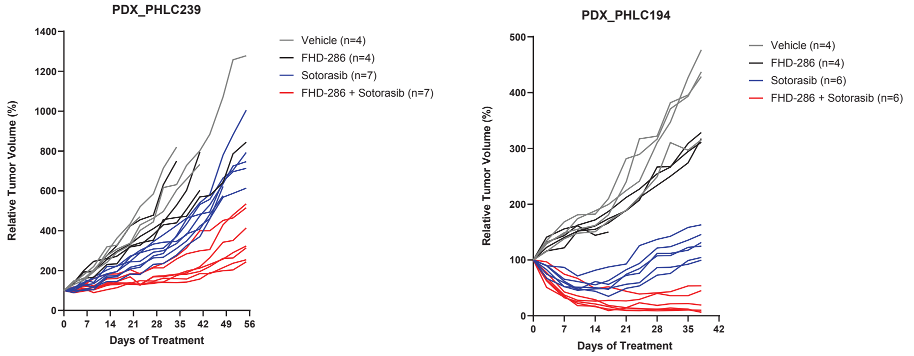

D

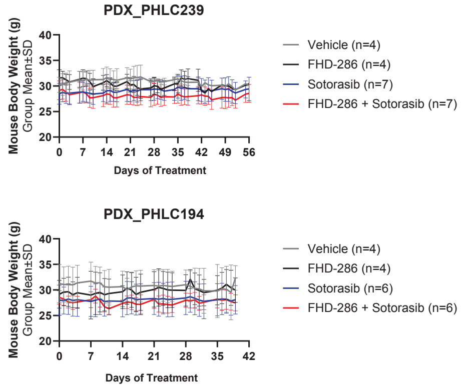

Figure S5. mSWI/SNF inhibition (FHD-286) sensitizes organoid and in vivo patient-derived xenograft models to sotorasib. A. Summary table of characteristics for xenograft-derived organoid models (XDOs) and patient-derived xenograft models (PDXs) used in this study. **B.** 3D CTG analysis for organoids, XDO_PHLC344 and XDO_PHLC207, following 14 days of the indicated drug treatments (viability relative to DMSO control). **C.** Individual tumor growth kinetics representative of relative tumor volume for PDX models PHLC239 and PHLC194. **D.** Line graphs of mouse body weight (in grams) across indicated drug treatments in PDX models PHLC239 and PHLC194. Data represent group mean +/- SEM.
